# Supplementary material for: Identification of photoperiod-induced specific miRNAs in the adrenal glands of Sunite sheep (Ovis aries)
Source: Front Vet Sci. 2022 Jul 22;9:888207. doi: 10.3389/fvets.2022.888207 (PMC9354845; doi:10.3389/fvets.2022.888207)
Supplement: Supplementary file 1 [file Data_Sheet_1.ZIP › Supplementary file/miRDeep2 file.zip!/miRDeep2 file/Novel_Structure/LP42A3/Novel_705.pdf]

Provisional ID	:	NC_019484.2_24697
Score total	:	23.9
Score for star read(s)	:	3.9
Score for read counts	:	16.4
Score for mfe	:	2
Score for randfold	:	1.6
Score for cons. seed	:	
Total read count	:	44
Mature read count	:	36
Loop read count	:	0
Star read count	:	8

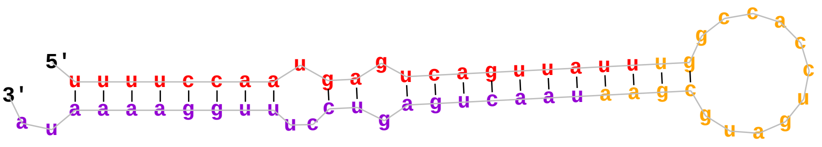

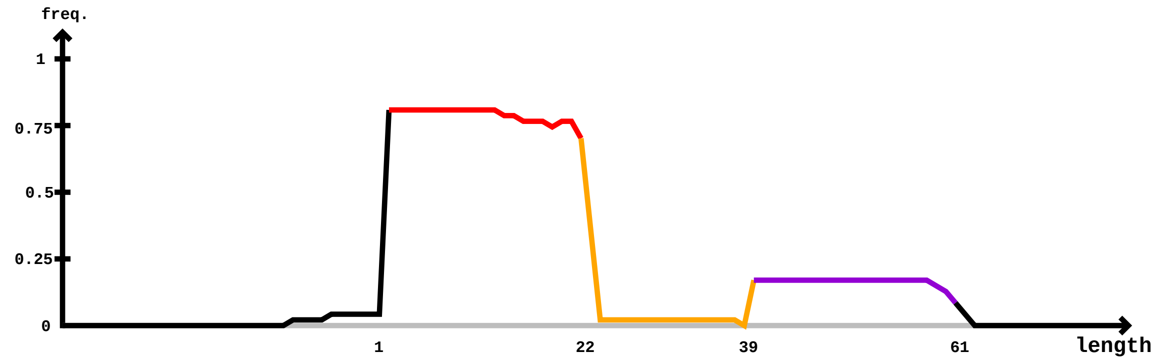


```
                                Mature                              Star
5'- ucucccuguuuuaaaacuucaacaucagggucuuuuccaaugagucaguuauuuggccaccugaugcgaauaacugaguccuuuggaaaauacccugaugcugggaaag  -3'   obs
    ucucccuguuuuaaaacuucaacaucagggucuuuuccaaugagucaguuauuuggccaccugaugcgaauaacugaguccuuuggaaaauacccugaugcugggaaag         exp
    ..((((.(((....))).....(((((((((.((((((((.((.(((((((((((...........))))))))))).))..)))))))).)))))))))..))))...   reads   mm   sample
    ......................caucagggucuuuucAaaugaguc...............................................................   1       1    seq
    ..........................agggucuuuuccaaugaA.................................................................   1       1    seq
    ................................uuuuccaaugagucagu............................................................   1       0    seq
    ................................uuuuccaaugagucaguuau.........................................................   3       0    seq
    ................................uuuuccaaugagucaguuauC........................................................   2       1    seq
    ................................uuuuAcaaugagucaguuauu........................................................   2       1    seq
    ................................uuuuccaaugagucaguuauu........................................................   22      0    seq
    ................................uuuuccaaugaAucaguuauu........................................................   1       1    seq
    ................................uuuuccaaugagucaguuauuA.......................................................   5       1    seq
    ..................................................Cuuuggccaccugaugcga........................................   1       1    seq
    ......................................................................uaacugaguccuuuggaaa....................   1       0    seq
    ......................................................................uaacugaguccuuuggaaaa...................   1       0    seq
    ......................................................................uaacugaguccuuuggaaaau..................   2       0    seq
    ......................................................................uaacugaguccuuuggaaaaua.................   4       0    seq
```
